# Improvement of palmoplantar pustulosis after excision of polyacrylamide injected into the nasal region

**DOI:** 10.1080/23320885.2021.2002155

**Published:** 2021-11-11

**Authors:** Kazuya Kashiyama, Jinyoung Lee, Kazufumi Koga, Yumi Matsuo, Katsumi Tanaka

**Affiliations:** Department of Plastic and Reconstructive Surgery, Nagasaki University Hospital, Nagasaki, Japan

**Keywords:** Polyacrylamide, autoimmune syndrome induced by adjuvants, polyacrylamide hydrogel

## Abstract

We report a patient in whom polyacrylamide hydrogel injected into the nasal region caused palmoplantar pustulosis. We report this case because few cases of autoimmune syndrome induced by adjuvants caused by polyacrylamide have been reported.

## Introduction

Non-biocompatible synthetic material may induce a local inflammatory reaction, migrate to distant organs, and activate the immune system. We report a patient in whom polyacrylamide injected into the nasal root region during rhinoplasty acted as an adjuvant and caused palmoplantar pustulosis.

## Case presentation

Patient: A 36-year-old female

Familial medical history: None

Past medical history: The patient received a local injection of polyacrylamide hydrogel (PAAG) into the nose root region at an aesthetic clinic to make her nose taller. 13 years later, she noted redness at the injection region and visited an aesthetic clinic again. She underwent incisional drainage and antibiotic administration, but no bacteria were detected on wound culture, which was aseptic. Wound culture was also performed multiple times thereafter, but no bacteria were detected in any of the cultures.

About 2 months after noticing redness in the nose root region, pustules developed on her palms and soles, so she visited a dermatology department and oral steroid treatment was initiated under a diagnosis of palmoplantar pustulosis. No local biopsy or serological tests were performed in making the diagnosis of palmoplantar pustulosis. She visited our department approximately 1 year after the onset of the symptoms to discuss the injected PAAG.

### Findings on the first examination

**Figure 1.**
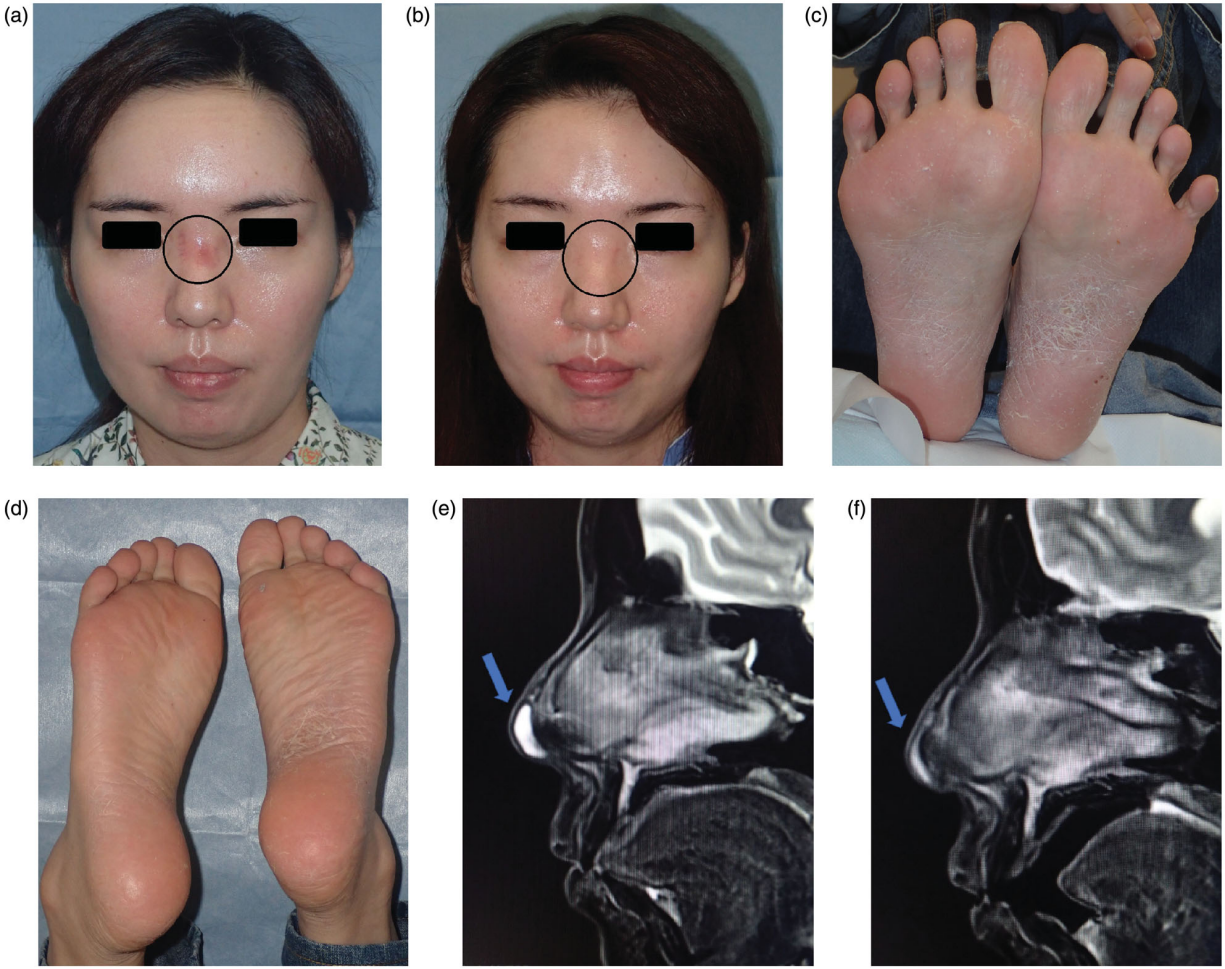
(a) Findings on the first examination: Redness and capillary dilatation were observed in the nose root region over the dorsum of the nose, indicated by the circle. (b) 1 year after surgery: Redness and capillary dilatation in the nose root region disappeared, indicated by the circle. (c) First examination at our hospital: Photograph of the sole. Pustules associated to scaling plaques and some fissures in the palmoplantar area were noted. (d) 1 year after surgery: Photograph of the sole. No recurrence of palmoplantar pustulosis was noted at 1 year after surgery. (e) MRI: A water-soluble implant remained in the region, indicated by the arrow. (f) 1 year after surgery: Postoperative reactions were noted in the region, indicated by the arrow.

Redness and the capillary dilatation were observed in the nose root region (Figure 1(a)). No wave or induration was palpated in this region. In addition, pustules associated with scaling plaques and some fissures in the palmoplantar area were noted (Figure 1(c)).

### Imaging findings

Subcutaneous retention of a water-soluble implant exhibiting high intensity on T2W1 was noted in the nasal apex (Figure 1(e)). The patient reported an abnormality in the injection region at the nose root, but this was not observed.

### Course at the first examination

**Figure 2.**
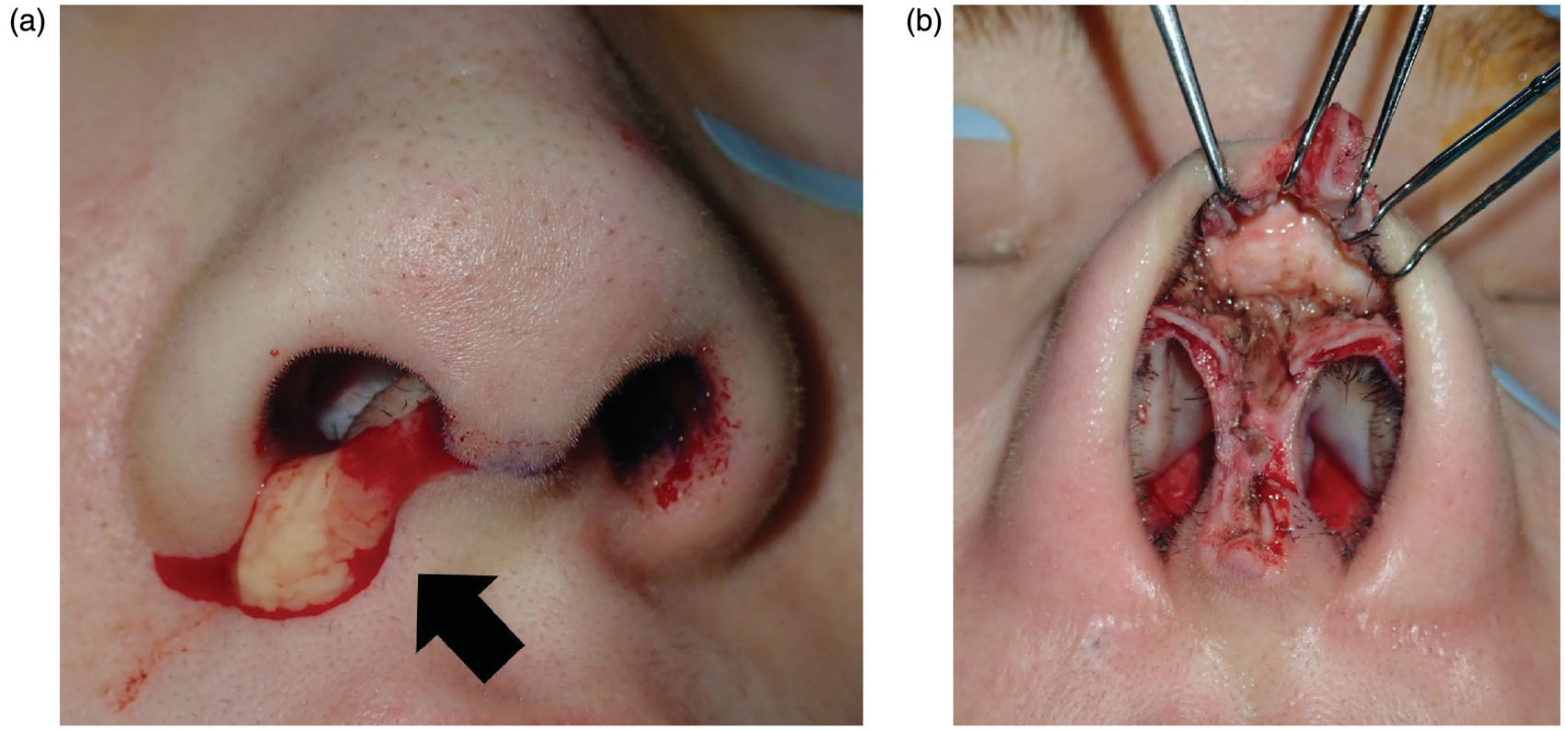
Intraoperative findings. (a) A water-soluble specimen in the wound region, indicated by the arrow. (b) At the completion of debridement.

**Figure 3.**
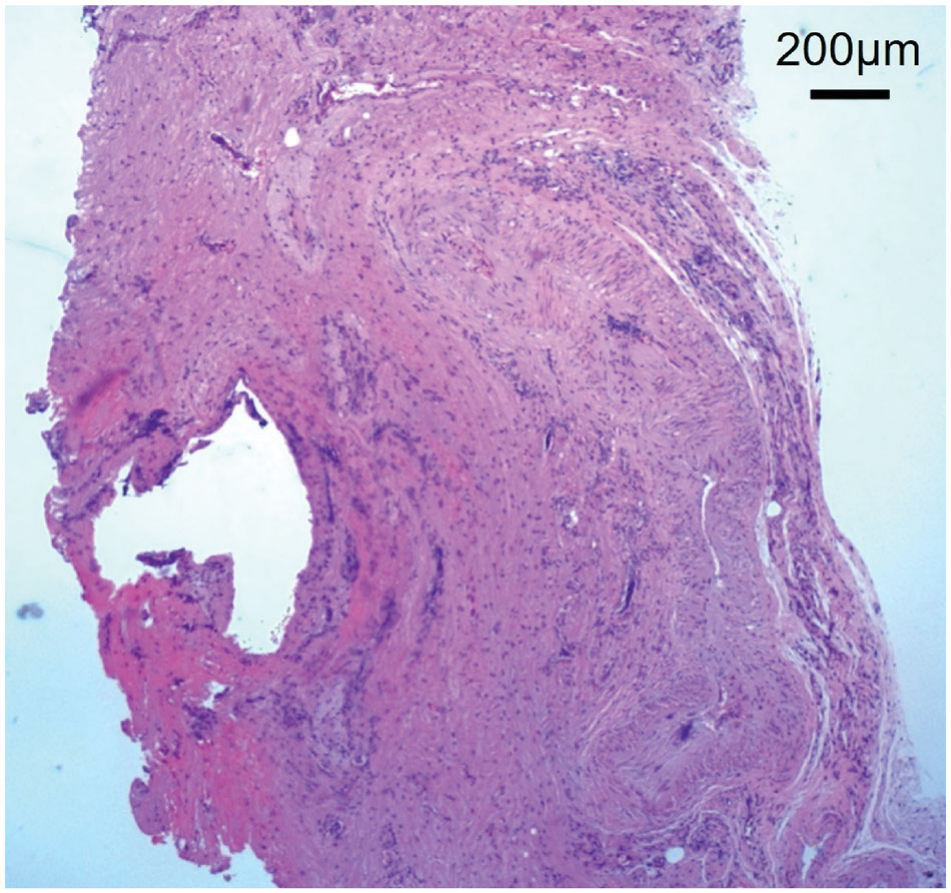
Histopathological view of granulation tissue: Inflammatory cell infiltration was observed. Scale bar 200 µm.

Implant excision and debridement of the nasal apex were performed under general anesthesia. The nasal apex was approached by open rhinoplasty. The wound region was filled with granulation tissue and a water-soluble implant (Figure 2(a)). After implant excision, the wound region was curetted, irrigated, and closed by suturing (Figure 2(b)). Methicillin-resistant coagulase-negative staphylococci (MRCNS) were detected in the culture of a fluid that leaked through the incision (Figure 2(a)). However, no bacteria were detected in a culture of the wound region excised from the back of the lesion. On pathological examination, granulation tissue infiltrated by inflammatory cells was observed (Figure 3). As palmoplantar pustulosis started to improve approximately 3 months after surgery, the steroid dose was gradually reduced and oral steroid treatment was discontinued 6 months after surgery. No recurrence of palmoplantar pustulosis was noted 1 year after surgery (Figure 1(d)). Redness and capillary dilatation of the nose root region spontaneously disappeared 1 year after surgery (Figure 1(b)). No abnormalities were noted in the surgical region of the nasal apex. On imaging, a postoperative reaction was observed in the surgical region 1 year after surgery (Figure 1(f)).

## Discussion

Miyoshi et al. reported in 1964 that a silicone breast implant placed in the body for a long time acted as an adjuvant and induced autoimmunity [1]. Later, Shiokawa et al. defined human adjuvant disease as an autoimmune disease induced by artificial materials [1]. In 2011, Shoenfeld & Agmon-Levin proposed a concept that vaccines and adjuvants induce autoimmune disease, termed autoimmune syndrome induced by adjuvants (ASIA) [2]. Recently, a lot of evidence for adjuvant-related autoimmune stimuli has been gathered [3,4]. In the plastic surgery/aesthetic surgery field, it was previously reported that silicone implants in the breasts may cause autoimmune disease as a complication [5]. We reported a patient in whom PAAG injected into the dorsum of the nose during rhinoplasty induced symptoms similar to palmoplantar pustulosis, and symptom remission was achieved with PAAG removal. In the past, these synthetic substances that were considered inert are currently causing complications in clinical practice. Today, there seems to be no consensus on how an immune response to non-biocompatible synthetic material develops. The theory behind the adjuvant autoimmune disease is controversial. There is a hypothesis that material migration to distant organs leads to the direct toxicity of adjuvants in these regions. These local reactions are exacerbated in an acute manner at the origin of the implant by macrophage-antigen inflammatory stimulation and activation of type II inflammatory response by lymphocytes and cytokines.

Palmoplantar pustulosis is considered a localized type of pustular psoriasis in Western countries, whereas it is treated as an independent disease in Japan. The incidence increases among those in their 40 s, and multiple aseptic pustules are formed on the palms and soles. It is treated by steroids and active-type vitamin D_3_ externally, but the mean duration of illness is 10 years or longer, and it is an intractable skin disease [6]. The following factors have been discussed as possible causes; tobacco smoke, focal infections, microbiome imbalance, stress, and metal allergies [7]. In our patient, as palmoplantar pustulosis improved after excision of polyacrylamide, it was judged that polyacrylamide acted as an adjuvant and caused the disease.

PAAG is a non-absorbable water-soluble gel. It is used in augmentation mammoplasty, rhinoplasty, and treatment of wrinkles, and is promoted as a relatively easy procedure that can be performed under local anesthesia. It does not require hospitalization, has a short downtime after surgery, does not require repeated injections because the substance is non-absorbable, unlike hyaluronic acid, and provides a natural appearance. It is composed of polyacrylamide (2–4%) and saline (96–98%), and is polymerized as a chain polymer to remove the biological membrane permeability of acrylamide to detoxify it. However, its safety is currently under discussion [8]. PAAG was introduced as a new material for augmentation mammaplasty around 2000 in China, but its manufacture, sale, and injection are now prohibited because of adverse effects. No major complications were observed over several years of observation in the early phase after its use for augmentation mammaplasty, but problems have been observed over long-term observation such as aberration of other regions, induration, infection, mastitis, and impairment of breastfeeding [9]. PAAG was reported to be safe because it is highly biocompatible and not carcinogenic, unlike another simple substance, Aquamid [10,11]. However, concerns about neurotoxicity and carcinogenicity have been frequently reported [9,12–14].

It is necessary to investigate the influence of the long-term presence of PAAG in the human body. In the present patient, PAAG was injected into the nose root region, and redness and the capillary dilatation were observed in the same region, but the implant remained in the nasal apex on MRI. Liquid implants, such as PAAG and Vaseline, may migrate in tissue after injection. In this patient, PAAG injected into the nose root region may have moved to the nasal apex over time. Although redness of the nose root region was observed on the first examination, only excision of the implant remaining in the nasal apex and debridement was performed because no abnormalities such as drainage were observed, and damage to the nose root region was of concern to the patient. After implant excision, redness of the nose root region gradually disappeared, and palmoplantar pustulosis gradually improved, allowing the discontinuation of steroid administration. The non-biocompatible synthetic material PAAG may induce a local inflammatory reaction, migrate to distant organs, and activate the immune system.

In the present case, wound culture was performed multiple times before palmoplantar pustulosis was diagnosed at the dermatology department and after initiating steroid treatment, but no bacteria were detected in any of the cultures, which were all aseptic. MRCNS was detected in the culture of a fluid that leaked through the incision during surgery at our department, but no bacteria were detected by the culture of a specimen collected from the back of the lesion. As MRCNS was also detected in nasal culture routinely performed before surgery, we considered that infection of the PAAG-retained region was not established and PAAG was the cause of the palmoplantar pustulosis. However, it is well known that focal infection is a cause of the development and aggravation of palmoplantar pustulosis [15,16]. Regarding the association between PAAG and autoimmune diseases like palmoplantar pustulosis, the immunohistological investigation is necessary in many cases to clarify a causal relationship.

## Summary

We report a patient in whom PAAG was injected to lift the nose may have induced palmoplantar pustulosis. Even a small amount of acrylamide used in rhinoplasty may cause ASIA, leading to symptoms similar to palmoplantar pustulosis.
